# Social Isolation and Associated Factors in Chinese Adults With Epilepsy: A Cross-Sectional Study

**DOI:** 10.3389/fneur.2021.813698

**Published:** 2022-01-11

**Authors:** Rui Zhong, Hanyu Zhang, Qingling Chen, Xin Guo, Yujian Han, Weihong Lin

**Affiliations:** ^1^Department of Neurology, The First Hospital of Jilin University, Changchun, China; ^2^Department of Hepatology, Second People's Clinical College of Tianjin Medical University, Tianjin, China

**Keywords:** social isolation, Berkman-Syme Social Network Index, epilepsy, depressive symptoms, quality of life

## Abstract

**Objective:** We aimed to determine the prevalence of social isolation and associated factors among adults with epilepsy in northeast China.

**Methods:** A cohort of consecutive patients with epilepsy (PWE) from the First Hospital of Jilin University (Changchun, China) was recruited. Demographic and clinical data for each patient were collected during a face-to-face interview. Social isolation was measured using the Berkman-Syme Social Network Index (SNI), and the Neurological Disorders Depression Inventory for Epilepsy (NDDI-E) and Quality of Life in Epilepsy Inventory (QOLIE-31) were also administered. Multivariate logistic regression analyses were used to determine the factors associated with social isolation in PWE.

**Results:** A total of 165 patients were included in the final analysis. The mean SNI score was 2.56 (SD: 1.19), and 35 patients (21.2%) were socially isolated. In multivariate logistic regression analysis, higher depressive symptom levels (OR = 1.15, 95% CI: 1.003–1.318, *P* = 0.045) and poorer quality of life (OR = 0.967, 95% CI: 0.935–0.999, *P* = 0.047) emerged as independent factors associated with social isolation in PWE.

**Conclusion:** Social isolation is common and occurs in approximately one-fifth of PWE. Social isolation is significantly associated with depressive symptoms and poor quality of life in PWE. Patients need to be encouraged to actively integrate with others and reduce social isolation, which may help improve their quality of life.

## Introduction

Epilepsy is one of the most common serious brain diseases and affects more than 70 million individuals worldwide (1). Epilepsy has a profound long-term impact on various aspects of social life for patients, such as social anxiety (2), poor social support (3), perceived stigma (4), psychosocial burden (5), poor social adjustment (6), impaired social cognition (7), and lack of social skills and competence (8). These negative social aspects may lead to social isolation, which has yet to be fully investigated in adults with epilepsy.

Social isolation was defined as an objective lack or limitation of close personal ties to friends and family and social ties to the community (9, 10). Social isolation has been reported to be related to poor physical health and psychiatric conditions such as depressive symptoms (11–13). Psychiatric disorders have been identified as the most common comorbidities and one of the most important determinants of poor quality of life in patients with epilepsy (PWE) (14). Thus, social isolation may be associated with poor quality of life in PWE. Social isolation has also been reported as a significant risk factor for reduced well-being, cognitive decline, and mortality in the general population (15, 16). A previous cross-sectional study from India showed that children with epilepsy had significant social deficits and that boys with epilepsy had limited social integration (17). These results may be due to perceptions of being overprotected by their parents (18).

Studies on the topic of social isolation in adults with epilepsy are limited (19). Thus, the current study aimed to investigate the prevalence of social isolation and to identify associated factors in adults with epilepsy in northeast China. It was expected that social isolation would be associated with depressive symptoms and low quality of life in patients.

## Methods

### Study Design and Participants

This research involved a cross-sectional study performed at The First Hospital of Jilin University, a large tertiary care hospital in Northeast China. Patients with a confirmed diagnosis of epilepsy (20) aged 18 years or older were invited to participate in the present study from May to July 2021. Participants were required to take a stable dose of antiseizure medications (ASMs) for at least 4 weeks. Participants were also required to have the physical, mental, and linguistic ability to complete self-report questionnaires and interviews adequately. We excluded individuals who had a severe brain disease other than epilepsy (e.g., dementia and Parkinson's disease), a serious physical disease (e.g., cancer, significant hepatic, or renal conditions), or psychiatric disorders (e.g., schizophrenia). Patients were also excluded if they were not willing to participate or did not provide written informed consent. The protocols of this study were approved by the ethics committee of our hospital, and each participant provided written informed consent stating his or her willingness to participate.

### Data Collection

Each participant had a face-to-face interview with one trained clinician (HZ) who collected the data uniformly. A structured questionnaire was employed to collect demographic, socioeconomic, and epilepsy-related data during the interview. The demographic and socioeconomic information included age, gender, marital status, employment status, and education level. The epilepsy-related variables consisted of age at onset, epilepsy duration, epilepsy type, seizure frequency during the last 6 months, and ASM therapy regimen. Patients with a high education level were those who had a university education or above. Additionally, patients were requested to complete a series of questionnaires during the interview at our hospital. The Berkman-Syme Social Network Index (SNI), Neurological Disorders Depression Inventory for Epilepsy (NDDI-E), and Quality of Life in Epilepsy Inventory (QOLIE-31) were employed to assess social isolation, depression, and quality of life in patients, respectively.

### Questionnaires

The Berkman-Syme SNI was used to measure social isolation in PWE (21). This measure assessed social networks and had four self-reported subcomponents: married (no = 0; yes = 1); close friends and relatives (0–2 friends and 0–2 relatives = 0; all other scores = 1); group participation (no = 0; yes = 1); and participation in religious meetings or services ( ≤ every few months = 0; >once or twice a month = 1). Social Network Index scores ranged from 0 to 4, and a lower SNI score represented a higher level of social isolation. Scores of 0 and 1 represented the socially isolated category.

The NDDI-E is a rapid and user-friendly questionnaire used to assess depressive symptoms in PWE during the past 2 weeks (22). Patients were requested to respond to six self-rated questions, and each item was rated on a four-point scale (1–4). The sum of the scores ranged from 6 to 24, and higher scores represented higher levels of depressive symptoms.

We measured quality of life using the QOLIE-31 inventory. The test consisted of 31 questions and seven subscales: (1) seizure worry, (2) overall quality of life, (3) emotional well-being, (4) energy/fatigue, (5) cognitive functioning, (6) antiepileptic medication effects, and (7) social functioning. The total score ranged from 0 to 100, with higher scores indicating a more favorable quality of life (23).

### Statistical Analysis

Categorical variables were described as numbers (percentages) and compared by chi-square tests or Fisher's exact tests. Continuous variables were expressed as the mean ± SD and analyzed by Student's *t*-test or Mann–Whitney U-tests depending on the normal or non-normal distribution of the data assessed via the Kolmogorov–Smirnov test. Additionally, Spearman correlation analyses were used to examine the relationships between SNI scores and continuous variables. Using multivariate logistic regression analyses, the independent variables associated with socially isolated categories were investigated. Variables with *p* < 0.05 in the univariate analyses were included in the multivariate stepwise logistic regression model. All data were analyzed using SPSS 26 (SPSS Inc., Chicago, IL, USA). All statistical tests were two-tailed, and *p* < 0.05 was considered statistically significant.

## Results

A total of 196 patients were invited to participate in the current study. Of that group, 31 patients were excluded for the following reasons: refusal to participate (11), inability to complete the questionnaires (*n* = 8), severe brain disease or other disorder (7), and <18 years old (*n* = 5). Thus, a total of 165 PWE were included in the study, with a median age of 33.88 years and a female percentage of 50.9%. The demographic and clinical characteristics of the total participants are shown in Table 1. Approximately half of the patients (48.5%) were married, and one-third of the patients (33.3%) were unemployed. The mean age at onset was 25.38 years (SD: 14.72), and the mean duration of epilepsy was 8.53 years (SD: 8.87). Focal epilepsy was the most common epilepsy type reported by 137 patients (83%). The mean SNI score was 2.56 (SD: 1.19), and 35 patients (21.2%) were socially isolated.

**Table 1:** Demographic and clinical characteristics of total participants (*N* = 165).

**Variable**	**Number (%) or mean ± SD**
Age (years)	33.88 ± 13.63
Female	84 (50.9)
Married	80 (48.5)
Unemployed	55 (33.3)
High education level	85 (51.5)
Age at onset (years)	25.48 ± 14.72
Epilepsy duration (Years)	8.53 ± 8.87
Epilepsy type	
Focal	137 (83.0)
Generalized	13 (7.9)
Unclassified	15 (9.1)
Seizure frequency in the last 6 months	
Seizure free	36 (21.8)
<1/month	89 (53.9)
≥1/month	40 (24.2)
ASM therapy regimen	
Monotherapy	115 (69.7)
Polytherapy	50 (30.3)
NDDI-E score	9.53 ± 3.94
QOLIE-31 score	49.92 ± 11.02
SNI score	2.56 ± 1.19
SNI score <2	35 (21.2)
SNI score ≥2	130 (78.8)

Patients in the socially isolated group were more likely to be younger (*p* = 0.024) and less likely to be married (*p* < 0.001) than those in the socially connected group. Polytherapy was more common in patients with social isolation (*p* = 0.008). Additionally, the NDDI-E score (*p* < 0.001) and QOLIE-31 score (*p* < 0.001) showed significant differences between patients in the socially isolated group and the socially connected group. There was no significant difference in gender, unemployment, high education level, age at onset, epilepsy duration, epilepsy type, or seizure frequency between the groups with, and without social isolation. For details, see Table 2.

**Table 2:** Comparison of demographic and clinical characteristics in socially isolated group and socially connected group.

**Variable**	**Number (%) or mean** **±** **SD**		
	**Socially** ** isolated group** ** (*n* = 35)**	**Socially** ** connected group** ** (*n* = 130)**	***p*-Value**
Age (years)	29.46 ± 11.54	35.08 ± 13.94	0.024
Female	19 (54.3)	65 (50.0)	0.653
Married	7 (20.0)	73 (56.2)	<0.001
Unemployed	12 (34.3)	43 (33.1)	0.893
High education level	18 (51.4)	67 (51.5)	0.991
Age at onset (years)	21.83 ± 11.16	26.47 ± 15.43	0.212
Epilepsy duration (Years)	7.89 ± 7.46	8.70 ± 9.23	0.856
Epilepsy type			
Focal	30 (85.7)	107 (82.3)	0.235
Generalized	1 (2.9)	12 (9.2)	
Unclassified	4 (11.4)	11 (8.5)	
Seizure frequency in the last 6 months			
Seizure free	5 (14.3)	31 (23.8)	0.217
<1/ month	18 (51.4)	71 (54.6)	
≥1/month	12 (34.3)	28 (21.5)	
ASM therapy regimen			
Monotherapy	18 (51.4)	97 (74.6)	0.008
Polytherapy	17 (48.6)	33 (25.4)	
NDDI-E score	12.60 ± 3.96	8.71 ± 3.52	<0.001
QOLIE-31 score	41.83 ± 10.36	52.09 ± 10.18	<0.001

The continuous variables that were significantly correlated with the SNI score are presented in Table 3. Age, age at onset, and QOLIE-31 score were positively correlated with SNI scores (*p* < 0.001, *p* = 0.001, and *p* < 0.001, respectively). Additionally, the NDDI-E score was negatively correlated with the SNI score (*p* < 0.001, *p* < 0.001, and *p* < 0.001, respectively). No correlation was found between epilepsy duration and SNI score.

**Table 3:** Correlation between SNI scores and the continuous variables in PWE.

**Variable**	**Berkman-Syme Social Network Index**	
	***r***	***p*-Values**
Age	0.327	<0.001
Age at onset	0.257	0.001
Epilepsy duration	0.007	0.929
NDDI-E score	−0.436	<0.001
QOLIE-31 score	0.358	<0.001

Table 4 shows the independent factors associated with social isolation among PWE. In a multivariate logistic regression analysis, social isolation (present/absent) was the dependent variable, while age, age at onset, ASM therapy regimen, NDDI-E score, and QOLIE-31 score were included as covariates in the model. Higher depressive symptom levels (OR = 1.15, 95% CI: 1.003–1.318, *P* = 0.045) and poorer quality of life (OR = 0.967, 95% CI: 0.935–0.999, *P* = 0.047) emerged as independent factors associated with social isolation in PWE. The adjusted odds for social isolation in PWE increased by a factor of 1.15 with each point of increase in NDDI-E depressive scores. The adjusted odds for social isolation in PWE was 0.967 times smaller with each point of increase in QOLIE-31 scores. For details, see Table 4.

**Table 4:** Logistic regression analysis of the associations of social isolation.

**Variable**	***P*-values**	**OR**	**95 % CI**	
			**Lower limit**	**Upper limit**
NDDI-E score	0.045	1.15	1.003	1.318
QOLIE-31 score	0.047	0.967	0.935	0.999

## Discussion

To our knowledge, this is the first study aimed at investigating the prevalence of social isolation and associated factors in adults with epilepsy. Findings from this study suggested that approximately one-fifth of patients are socially isolated, and that depressive symptoms and poor quality of life are independent factors associated with social isolation in PWE.

The prevalence of social isolation has been previously reported to range from 20 to 67.5% in PWE (19, 24). The 68% of PWE with social isolation reported by a recent cross-sectional study from USE was higher than the 21.2% of PWE reported in the current study (19). A prospective observational study reported that the prevalence of social isolation was 20% in children with symptomatic generalized epilepsy (24). One possible explanation for these results could be the differences in the basic characteristics of patients across studies. Another explanation for the different prevalence of social isolation might be cross-cultural differences.

This study found that marital status was associated with social isolation in PWE, and married patients were less likely to be socially isolated. Marital relationships are an important component of family support. Married adults reported more family support and fewer depressive symptoms (25). Patients with epilepsy without family support had poorer seizure control and a higher lifetime prevalence of psychiatric disorders than those who had family support (26). However, it remains uncertain whether PWE who lived with other people in the same household experienced less social isolation. A recent study from Japan also showed that during the nationwide COVID-19 pandemic, PWE who lived alone were more likely to experience increased seizure frequency, indicating the relationship between seizure control and social connection in patients (27). However, adults with epilepsy were more likely to be unmarried throughout their lives due to social discrimination and stigma (28, 29). Families continue to object to their children marrying PWE. Undesirable marital status may lead to a lack of family support and social isolation in patients.

We found that age and age at onset were also associated with social isolation in PWE. However, this significant association disappeared in multivariate analysis. Patients with social isolation were more likely to be younger and to have had an earlier age at first seizure onset. This finding was in agreement with prior investigations (17, 30). The parents of children with epilepsy said that friends and relatives who knew that the child had epilepsy always treated the child differently and did not like to be left alone with the child (30). Pal et al. similarly reported that children with epilepsy tended to have limited social interaction compared with their peers but that they spent more time interacting with peer groups as they grew older (17).

In the current study, we found that the adjusted odds for social isolation in PWE increased by a factor of 1.15 with each point of increase in NDDI-E depressive scores. Socially isolated young adults were more likely to have depression, showing the beneficial role of social relationships in mental health (31). However, our finding was not supported by a recent study (19). Stauder and his colleague reported that social integration was not associated with depressive burden in PWE (19). One possible explanation for these controversial findings is the relatively small sample size in their cohort. Another explanation for the results may be that the instrument employed to assess depressive symptoms differed between the studies. It is well-known that the PHQ-9 instrument has a broader range of scores than the NDDI-E (0–27) (4). The NDDI-E is specific to the population with epilepsy. Patients with epilepsy feel stigmatized and subject to discrimination if they have a seizure in front of others, which leads to limited social interaction and further exacerbates depression (32, 33). Social isolation acts as a stressor that leads to alterations in reactivity to stress and social behavior in people (34). It has been reported that stressful life events (e.g., divorce of parents or loss of a child) were a significant predictor of anxiety and depression in the general population (35). Additionally, the adjusted odds for social isolation in PWE decreased by a factor of 0.967 with each point of increase in QOLIE-31 scores, indicating the relationship between social isolation and poor quality of life in PWE. A prospective observational study similarly reported that social isolation has detrimental effects on the quality of life in older adults (36). Adolescents with epilepsy feel that their greatest interpersonal challenge is forming relationships with others, which may have a negative effect on their quality of life (37). It is well-known that social isolation is the lower end of a distribution of social support. Adults with epilepsy who lacked affectionate support were significantly more likely to report poor quality of life (38). Thus, PWE needs to be encouraged to actively integrate with others and reduce social isolation, which may help improve their quality of life.

This study faces several limitations. First, the cross-sectional design greatly limits the ability to draw any inferences regarding the causal relationship between these variables and social isolation, and regression analyses in a cross-sectional design study also have limited results. Second, the sample size in the socially isolated subgroup was relatively small, and all participants were adults with epilepsy enrolled from a large tertiary care hospital in northeast China. Thus, it cannot be claimed that this sample is representative of Chinese PWE. Third, the use of self-report for all measures in the present study may allow for response bias. Additionally, some variables that may be associated with social isolation in epilepsy (e.g., AED type) were not available and were not analyzed for our cohort. Finally, this study was performed during the COVID-19 pandemic. Social isolation and psychiatric symptoms may have been more frequent in PWE during this period.

## Conclusion

The findings suggest that social isolation is common in adults with epilepsy. Social isolation is associated with depressive symptoms and poor quality of life. Further studies should examine whether improvements in social connection can improve depressive symptoms and quality of life in these patients.

## Data Availability Statement

The raw data supporting the conclusions of this article will be made available by the authors, without undue reservation.

## Ethics Statement

The studies involving human participants were reviewed and approved by Ethics Committee of First Hospital, Jilin University. The patients/participants provided their written informed consent to participate in this study.

## Author Contributions

RZ and WL conceived of and designed the study. RZ, HZ, XG, and YH were involved in data acquisition. RZ and QC analyzed the data and wrote the manuscript. All authors contributed to the article and approved the submitted version.

## Funding

This work was supported by a grant from the Programme of Jilin University First Hospital Clinical Cultivation Fund (LCPYJJ2017006).

## Conflict of Interest

The authors declare that the research was conducted in the absence of any commercial or financial relationships that could be construed as a potential conflict of interest.

## Publisher's Note

All claims expressed in this article are solely those of the authors and do not necessarily represent those of their affiliated organizations, or those of the publisher, the editors and the reviewers. Any product that may be evaluated in this article, or claim that may be made by its manufacturer, is not guaranteed or endorsed by the publisher.
